# Neighbourhood Walkability and Physical Activity during the COVID-19 Pandemic

**DOI:** 10.3390/ijerph21040387

**Published:** 2024-03-22

**Authors:** Sigit D. Arifwidodo, Orana Chandrasiri

**Affiliations:** 1 Department of Landscape Architecture, Faculty of Architecture, Kasetsart University, Chatuchak 10900, Thailand; 2 Activethai.org Research Center, Faculty of Architecture, Kasetsart University Chatuchak 10900, Thailand; orana.chandrasiri@gmail.com

**Keywords:** physical activity, neighbourhood walkability, green open spaces, COVID-19, built environment, landscape architecture

## Abstract

This study investigated whether living in a walkable neighbourhood could mitigate the adverse effects of the lockdown and closure of public open spaces during the COVID-19 pandemic on physical activity among adults in Bangkok, Thailand. We conducted a telephone survey with 579 respondents and collected information on their physical activity, access to green open spaces, neighbourhood walkability, and socioeconomic characteristics during the pandemic. Our study indicates that living in a walkable neighbourhood is associated with a higher likelihood of engaging in sufficient physical activity during the pandemic. Furthermore, we confirm the influence of socioeconomic factors and health behaviours on physical activity levels, aligning with previous research. Notably, our study highlights the significant association between access to green open spaces during lockdown and increased physical activity. These results underscore the importance of promoting walkable neighbourhoods and ensuring accessible green spaces to enhance physical activity and improve health outcomes during and beyond the pandemic.

## 1. Introduction

Regular physical activity provides multiple physical and mental health benefits. Evidence has found that physical activity is influenced not only by personal factors, such as motivation and confidence, but also by external factors, such as access to neighbourhood environments and exercise opportunities [1]. Various aspects of the neighbourhood environment, such as the availability of green open spaces near homes, can influence physical activity and health outcomes. In particular, living in a green neighbourhood can reduce mental stress, improve physical health, and reduce chronic disease risks. In urban areas, where space is limited, urban green spaces play an essential role in providing opportunities to increase the physical activity of the urban populations.

Additionally, there are positive associations between recreational physical activity and factors such as proximity, accessibility, size, quantity, and quality of urban green spaces [2]. Self-reported and objective measures of neighbourhood walkability, such as street and sidewalk connectivity, residential density, proximity, the mix of destinations and land uses, and pedestrian infrastructure, are consistently associated with physical activity [3,4]. Respondents who lived in neighbourhoods that were easy and safe for residents to walk from their homes to nearby amenities, such as grocery stores, schools, parks, and entertainment venues, were associated with higher levels of physical activity [5]. This is because these walkable neighbourhoods have features such as sidewalks, well-maintained streets, and pedestrian crossings that make walking more convenient and safer [6]. Additionally, walkable neighbourhoods often have amenities such as parks, shops, and restaurants in close proximity, which further encourages walking.

The COVID-19 pandemic has greatly impacted our daily routines, including physical activity [7]. Studies on the effects of COVID-19 on physical activity have shown that lockdowns and limited mobility are associated with decreased physical activity and increased sedentary behaviour [8]. Public health measures, such as social distancing and the closure of places, such as parks and recreational centres, have had a negative impact on people’s ability to engage in physical activity and healthy lifestyles [9]. Working from home and studying remotely have also reduced the amount of walking and physical activity that people typically engage in on a daily basis [10]. Lockdown measures have been associated with a significant reduction in physical activity levels, especially in older adults, those with lower socioeconomic status, and those with pre-existing health conditions [11]. Recent developments in this topic have shown that the neighbourhood environment has significantly cushioned decreased physical activity during the pandemic. For instance, limited access to urban green spaces near homes is a significant factor in decreased physical activity levels [12]. A neighbourhood with safe and accessible walking paths, parks, and outdoor spaces allows individuals to engage in physical activity while maintaining social distancing measures [13,14,15,16].

From March 2020 to July 2020, Thailand experienced a surge in the number of COVID-19 cases. The first case was reported on 13 January 2020, and by March 2020, the country had over 100 confirmed cases. The number of cases continued to increase, with the highest daily increase of 188 cases recorded on 22 March 2020. However, the country’s proactive response helped to flatten the curve and reduce the number of new cases. By the end of July 2020, Thailand had recorded 3304 cases and 58 deaths [17]. The government implemented strict measures to contain the spread of the virus, such as travel restrictions, curfews and lockdowns, and mandatory face masks [18]. These measures have resulted in a significant decrease in physical activity among individuals [19]. People have fewer physical activity options with closed gyms and parks and limited outdoor activities [20].

Despite a growing number of studies exploring the relationship between neighbourhood walkability and physical activity, the extent to which neighbourhood walkability contributes to increased physical activity levels or helps alleviate insufficient physical activity during the COVID-19 pandemic in Thailand has not been fully investigated. To address these gaps in knowledge, this study sought to examine the association between neighbourhood walkability and physical activity among adults in Bangkok, Thailand, during the COVID-19 pandemic. The primary research question guiding this study is “To what extent does living in a walkable neighbourhood help counteract the decline in physical activity levels caused by the lockdown measures and closure of public open spaces during the COVID-19 pandemic among adults in Bangkok, Thailand?” Our main hypothesis was that living in a walkable neighbourhood is associated with better physical activity levels. By focusing on this hypothesis, our study aimed to provide novel insights into the role of neighbourhood walkability in promoting physical activity under challenging circumstances. The findings of this study will contribute to the existing literature and offer valuable insights for policymakers and stakeholders to understand the importance of the neighbourhood environment in promoting physical activity, both during and after the pandemic.

## 2. Materials and Methods

This cross-sectional study was conducted during the lockdown period from March to May 2020. During this time, the national government implemented travel restrictions, curfews, and closures of public spaces for mass gatherings, such as public parks and plazas. Due to these measures, conducting face-to-face interviews was not possible in Bangkok. The data for this study were collected through a telephone survey, which was chosen to achieve more reliable sample distributions compared to an online questionnaire. The sample frame for this study was derived from the survey conducted in 2019 on public parks and physical activity in Bangkok.

In 2019, a survey focusing on urban green spaces and physical activity in Bangkok was conducted. Utilising a quota sampling methodology, the city was segmented into administrative districts for the collection of primary data. The Bangkok Metropolitan Administration (BMA) provided a database of registered households that served as the sampling framework. From this list, 12 adult individuals were randomly chosen from each of the 50 districts, amounting to a total of 600 respondents. To ensure comprehensive participation, ten surveyors were engaged to conduct in-person interviews, achieving a response rate of 100%. Respondents were compensated with a small gift valued at 100 Thai baht (approximately 30 US dollars) for their time. Additionally, with permission, contact numbers were collected for a subsequent telephone survey. The details on the sampling selection can be found in a separate publication [21].

We then used this list to conduct a telephone survey for this study, which was carried out in April 2020 by ten trained surveyors. The participants were preliminarily briefed on the study’s goals and processes before their consent was obtained. Following their participation, they received 100 Thai baht as a gesture of appreciation. The survey comprised structured questions across four key areas: (1) physical activity, (2) neighbourhood walkability, (3) ability to access green spaces near home during the lockdown, and (4) sociodemographic variables. To verify the reliability of the survey instrument, Cronbach’s alpha was employed, revealing an internal consistency rating of between 0.79 and 0.84. Post-data cleansing, 579 responses were deemed suitable for the analysis. In accordance with the ethical guidelines of the Declaration of Helsinki, this study was approved by the Institutional Review Board of the Institute for the Development of Human Research Protection (IHRP) in Thailand. Other findings from this survey can also be found in a separate publication [22]. The following paragraphs describe the variables used in this study.

The physical activity level was assessed using the Global Physical Activity Questionnaire (GPAQ), which quantifies the amount of physical activity (in minutes per week) in the work, transportation, and recreation domains. The GPAQ was validated and interviewer-administered and adapted to the Thai context following previous studies in Thailand [21,22,23]. According to the 2020 World Health Organization (WHO) global guidelines on physical activity for health, it is recommended that adults aged 18–64 years should engage in 150–300 min of moderate-intensity aerobic physical activity weekly [24]. Based on this guideline, we categorised the responses from the Global Physical Activity Questionnaire (GPAQ) into two groups: those who had sufficient physical activity (meeting the physical activity recommendation of 150 min or more per week) and those with insufficient physical activity (not meeting the physical activity recommendation with less than 150 min per week).

We also gathered other individual data, such as health behaviours (the presence of non-communicable diseases (NCDs), smoking and alcohol consumption habits, and body mass index (BMI)). These variables have been acknowledged in the literature to be associated with physical activity [25,26]. We also constructed a variable for whether the respondents had children or dogs with dichotomised “yes/no” responses, following previous studies [27,28]. Having children or dogs in the household was associated with having higher physical activity levels both in children and adults [29]. Finally, we also collected sociodemographic variables such as income, education, gender, marital status, and occupations of the respondents.

The present study obtained two proxy variables concerning neighbourhood walkability. The first measure employed was the Neighbourhood Environment Walkability Scale-Abbreviated (NEWS-A), which is a validated tool for evaluating walkability as it encompasses multiple sub-scales that account for various aspects of physical activity and the built environment. The NEWS-A measures eight sub-scales: (1) land-use mix diversity, (2) land-use mix access, (3) street connectivity, (4) walking/cycling facilities, (5) aesthetics, (6) safety from traffic, (7) safety from crime, and (8) residential density. All sub-scales were measured on a 5-point Likert Scale (1 = strongly disagree to 5 = strongly agree) following a previous study [30]. To calculate the sub-scale scores, the responses to each item within a sub-scale were summed and divided by the number of items in that sub-scale. A similar method was used to calculate the NEWS-A aggregate score, where lower scores represented lower walkability, and higher scores represented higher walkability [31]. For the analysis, we then dichotomised the NEWS-A scores into “high walkability” and “low walkability”. Some question items were modified to reflect the Thai context and ensure that the telephone survey length was manageable. The reliability and validity of the NEWS-A have been previously shown in several countries, with all included scales having test–retest reliability intraclass correlations of >0.50 [32]. The NEWS-A variable in this study had an acceptable internal consistency (Cronbach’s α = 0.83).

The second proxy variable captured access to green spaces near homes suitable for physical activity during the lockdown period. This variable was considered because the NEWS-A could not capture the lockdown situation in which all the public spaces for physical activity were closed. Therefore, we included it as a separate variable to provide a comprehensive assessment of neighbourhood walkability during the COVID-19 pandemic. We asked whether the respondents could access the green open spaces near home (around 10–15 min walking or around 1 km), such as community areas, gardens, plazas, or closed streets that could be used for physical activity during the lockdown, with “yes” and “no” as the possible responses. A previous study showed that access to green open spaces during the COVID-19 pandemic could cushion the physical activity reduction in the urban population [33]. Hypothetically, limited access to these green open spaces near home for physical activity during the lockdown could cause a significant reduction in physical activity levels.

For data analysis, frequencies and means were used to summarise the socioeconomic and neighbourhood environmental characteristics. We utilised a multivariable logistic regression model (odds ratio [ORs], 95% confidence interval [CI]) to examine the relationship between physical activity level and the independent variables. The model was adjusted for potential confounding factors. All data analyses were conducted using IBM SPSS Statistics 24.

## 3. Results

Table 1 presents the descriptive statistics of the sample, which consisted of 579 respondents. The sample comprised a majority of females (56.0%), unmarried individuals (59.6%), and those with a monthly income in the range of 300–1000 USD per month (46.1%). The proportion of respondents with post-graduate education was only 5.8%. More than 10% of the respondents reported regular alcohol consumption, while 10% reported regular smoking. Most respondents (90%) reported not having any NCDs. A substantial proportion of the respondents (62.9%) reported inadequate levels of physical activity (i.e., engaging in 150 min or less of physical activity). As shown in Table 1, only 37.1% of the respondents lived in a walkable neighbourhood. Most respondents also reported they did not have access to green spaces during the lockdown (60.3%).

Table 2 shows the association between neighbourhood walkability and physical activity among adults during the COVID-19 pandemic. The findings indicated that individuals residing in walkable neighbourhoods were significantly more likely to engage in sufficient physical activity during the pandemic (OR = 5.689, *p* < 0.005). In addition, respondents who had access to green open spaces during the lockdown were twice as likely to meet the physical activity guidelines recommended by the World Health Organization (OR = 2.504, *p* < 0.005). Healthy behaviours, such as abstaining from regular alcohol consumption (OR = 1.382, *p* < 0.005) and smoking (OR = 3.938, *p* < 0.005), were positively linked to sufficient physical activity. Conversely, individuals with a BMI of over 25 were more likely to engage in insufficient physical activity. Moreover, only several sociodemographic characteristics of the respondents were significantly associated with sufficient physical activity. Higher education was associated with a higher probability of having sufficient physical activity (high school to bachelor’s degree OR = 2.105, *p* < 0.005; more than bachelor’s degree OR = 8.194, *p* < 0.005). Women were found to be considerably less likely than men to engage in adequate levels of physical activity (OR = 0.4999, *p* < 0.005).

## 4. Discussion

This study examined the association between neighbourhood walkability and physical activity during the COVID-19 pandemic. This study has two major findings. First, the respondents’ healthy behaviours and sociodemographic variables were significantly associated with sufficient physical activity during the pandemic. Respondents with higher education were more likely to engage in sufficient physical activity. Previous studies have also found a similar result, stating that this may be due to a greater awareness of the benefits of physical activity and access to resources, such as exercise programs and facilities, during the pandemic [34,35]. In our study, being female was associated with a lower likelihood of having sufficient physical activity. Some studies have found that the pandemic has reduced physical activity levels in both genders [36,37]. Other studies have found that women are more likely to experience post-COVID-19 symptoms that affect their physical activity levels [38,39]. One of the explanations is that the varying circumstances surrounding governmental interventions have led to differing levels of COVID-19 exposure among individuals. Further studies are required to investigate the complex relationship between sex and physical activity levels during the pandemic.

We found that the healthy behaviours of the respondents were significantly associated with physical activity. The likelihood of having sufficient physical activity was reduced among those who consumed alcohol regularly during the pandemic. Previous studies have shown similar results, indicating that the closure of bars and restaurants has led to increased alcohol consumption at home, which has negatively impacted physical activity levels [40,41]. Non-smokers were more likely to engage in sufficient physical activity during the pandemic. Previous research has linked regular smoking with reduced physical activity levels, and the COVID-19 pandemic has raised concerns among smokers about exacerbating their respiratory symptoms, potentially leading to the avoidance of physical activity [42]. The stress and anxiety associated with the pandemic may also increase smoking frequency and other sedentary behaviours, further reducing physical activity levels [43]. The presence of non-communicable diseases (NCDs) was negatively associated with sufficient physical activity. Individuals with pre-existing NCDs may avoid physical activity because of concerns about exacerbating their symptoms or increasing their risk of contracting the virus. Conversely, reduced physical activity levels during the pandemic may have increased the risk of developing NCDs. Inactive individuals may be at greater risk of developing NCDs owing to a sedentary lifestyle, poor diet, and pandemic-related stress [44,45,46].

The second major finding of this study highlights a substantial link between neighbourhood walkability and physical activity. Specifically, individuals residing in high-score walkable neighbourhoods were almost six times more likely to engage in sufficient physical activity during the pandemic. This finding is consistent with prior research, which has shown that residents of walkable neighbourhoods are more physically active during lockdowns than those residing in less walkable areas [47,48]. The design of compact and walkable neighbourhoods has been found to promote physical activity and reduce the risk of obesity-related chronic diseases, such as diabetes and heart disease, which could increase the severity of COVID-19 infection and mortality risk [49]. Additionally, walkable neighbourhoods consistently lower the prevalence of and deaths associated with COVID-19 [50]. Another study suggested that walkable neighbourhoods may have helped to mitigate the negative impact of lockdowns on eating behaviour and physical activity levels [51]. This finding reiterates the argument that a walkable neighbourhood is an essential determinant in creating a built environment conducive to physical activity and a healthy lifestyle, even during times of restricted movement.

Our study also revealed that individuals with access to green spaces near their homes during the lockdown were more likely to meet the requirements for sufficient physical activity. Previous studies also suggested similar findings that such individuals were more likely to engage in physical activity during the pandemic [52]. Green spaces offer a secure and easily accessible environment for activities such as walking, cycling, and jogging during the pandemic, contributing to the promotion of physical activity. In addition, green spaces support mental health outcomes such as reduced stress, anxiety, and depression [53,54]. A study in Canada found that children and youth experienced decreased outdoor activities during the pandemic [55]. In the UK and Germany, people spent more time in green spaces during the pandemic, considering them safe for socialising, physical activity, and recreation [56,57]. In Japan, there was a significant increase in pedestrian activities along the streets, city parks, peri-urban forests, and protected areas during the pandemic [58]. These studies highlight that green spaces can be visited safely during the pandemic. They may contribute to physical and mental well-being by providing opportunities for physical activity, social contact, and stress reduction. However, context-specific measures should be considered to ensure the safety of visitors. Research has also shown that inequalities in access to green spaces have been exacerbated during the pandemic [59]. The pandemic’s closure of parks and green spaces has narrowed the options for physical activity, which may impact vulnerable populations more than others. These limitations are particularly felt by low-income communities that have fewer opportunities to access green spaces, a situation that is compounded by the fact that these communities are often the hardest hit by COVID-19 [60]. Research has revealed disparities in the use and experience of green spaces, underlining the need to guarantee equal access to green spaces for all individuals, especially during a pandemic [61,62,63].

This study has several limitations that should be considered when interpreting the results. Firstly, the study’s cross-sectional design makes it impossible to establish a direct causal relationship between living in a walkable neighbourhood and an increase in physical activity levels. A more robust research method, such as a longitudinal survey with larger sample sizes and more rigorous probabilistic sampling, is necessary to establish causality. Secondly, this study acknowledges the potential for reverse causality, whereby more physically active individuals may seek out walkable neighbourhoods, as they may be predisposed to walking or cycling. The ongoing COVID-19 pandemic has further exacerbated this limitation, with lockdown measures reducing physical activity options for those living in non-walkable neighbourhoods. In addition to that, living in a walkable community is often challenging for families with limited economic resources. Walkable communities typically require a higher socioeconomic status, as they offer well-connected pedestrian infrastructure, access to amenities, and proximity to employment opportunities. However, the associated higher housing costs and increased cost of living can make it difficult for economically disadvantaged families to afford housing in these areas. Limited access to affordable housing and higher financial demands can restrict their ability to reside in walkable communities, depriving them of the benefits and opportunities offered by their walkability. Addressing these economic disparities is crucial to ensuring equitable access to walkable neighbourhoods for families with poor economic conditions.

Finally, the study’s reliance on self-reported physical activity and perceived walkability measures may have introduced measurement bias. These subjective measures may not accurately reflect the actual physical activity levels or objective walkability measures. Using more objective measures, such as GPS tracking and street audits, could help address these limitations and provide a more accurate assessment of physical activity and walkability.

## 5. Conclusions

This study explored the following research question: “Does neighbourhood walkability mitigate the negative impact of lockdown and closure of public open spaces during the COVID-19 pandemic on physical activity levels among adults in Bangkok, Thailand?” Our study revealed that living in a walkable neighbourhood was significantly associated with the attainment of sufficient physical activity during the pandemic. Additionally, our findings demonstrated consistent associations between socioeconomic variables, health behaviours, and the achievement of adequate physical activity, aligning with the existing literature even prior to the pandemic. Furthermore, we observed a significant relationship between access to green open spaces and physical activity during the lockdown period. These findings collectively indicate that living in a walkable neighbourhood is a crucial determinant in promoting physical activity, particularly when movement is restricted.

Although this study has certain limitations, including its cross-sectional design and reliance on self-reported measures, our results add to the expanding body of research highlighting the significance of neighbourhood walkability in promoting physical activity during the pandemic. Future research should aim to investigate the complex relationship between the neighbourhood environment and physical activity levels during the pandemic and use more objective measures to assess physical activity and walkability. It is also essential to conduct research that specifically targets populations, such as individuals with chronic conditions and the elderly. By focusing on these specific groups, a more comprehensive picture can be drawn regarding the impact of walkability on physical activity levels in the context of the pandemic. This targeted research would provide valuable insights into the potential challenges, barriers, and facilitators faced by these populations in engaging in physical activity within walkable neighbourhoods during this unique period. Such studies would contribute to a more nuanced understanding of the interplay between neighbourhood walkability, physical activity, and health outcomes, allowing for the development of tailored interventions and strategies to promote active lifestyles in diverse population segments. Ultimately, these findings have important implications for urban planning and public health policy, highlighting the need to prioritise the creation of walkable neighbourhoods and accessible green spaces to promote physical activity and improve health outcomes during and beyond the pandemic.

## Figures and Tables

**Table 1:** Respondents’ characteristics.

Category	Variable	Sample Characteristics
Sociodemographic characteristics	Monthly income	
	Less than 5000 THB (less than 160 USD)	30.1%
	5000–10,000 THB (160–300 USD)	8.3%
	10,001–30,000 THB (300–1000 USD)	46.1%
	30,001–50,000 THB (1000–1600 USD)	9.5%
	More than 50,000 THB	6.0%
	Education	
	High school or less	47.0%
	High school to bachelor’s degree	47.2%
	More than a bachelor’s degree	5.8%
	Gender	
	Male	44.0%
	Female	56.0%
	Marital status	
	Single	59.6%
	Living with partner	40.4%
	BMI	
	25 or less	77.0%
	>25	23.0%
	Occupation	
	Office-based workers	35.8%
	Labour-intensive workers	54.4%
	Unemployed/retired	9.8%
	Having children/dogs	
	Yes	38.9%
	No	61.1%
Healthy behaviour	Regular alcohol consumption	
	Yes	11.4%
	No	88.6%
	Regular smokers	
	Yes	10.0%
	No	90.0%
	Having non-communicable diseases (NCDs)	
	Yes	10%
	No	90%
Neighbourhood walkability	NEWS-A score	
	High	37.1%
	Low	62.9%
	Can access green spaces near home during lockdown	
	Yes	39.7%
	No	60.3%
Physical activity	Physical activity level	
	Sufficient physical activity (more than 150 min/week)	41.3%
	Insufficient physical activity (150 min/week or less)	58.7%

**Table 2:** Association between neighbourhood walkability and physical activity during the COVID-19 pandemic.

Category	Variable	OR	95%CI
Socioeconomic characteristics	Monthly income		
	Less than 5000 THB (less than 160 USD)	ref	
	5000–10,000 THB (160–300 USD)	1.362	0.618–3.002
	10,001–30,000 THB (300–1000 USD)	1.479	0.578–3.217
	30,001–50,000 THB (1000–1600 USD)	1.104	0.364–3.348
	Education		
	High school or less	ref	
	High school to bachelor’s degree	2.103 *	1.317–3.358
	More than a bachelor’s degree	8.431 *	2.450–29.011
	Gender		
	Male	ref	
	Female	0.499 *	0.315– 0.789
	Marital Status		
	Single	ref	
	Living with partner	1.389	0.819–2.355
	BMI		
	25 or less	ref	
	>25	0.475 *	0.291–0.776
	Occupation		
	Office-based workers	ref	
	Labour-intensive workers	1.087	0.602–1.964
	Unemployed/retired	0.608	0.578–3.935
	Having children/dogs		
	Yes	ref	
	No	0.796	0.511–1.240
Healthy behaviour	Regular alcohol consumption		
	Yes	ref	
	No	1.370 *	1.164–1.833
	Regular smokers		
	Yes	ref	
	No	3.882 *	1.803–8.361
	Having non-communicable diseases (NCDs)		
	Yes	ref	
	No	1.974 *	1.880–4.428
Neighbourhood environment	Neighbourhood walkability		
	Low	ref	
	High	5.735 *	3.751–8.678
	Access to green spaces near home during lockdown		
	No	ref	
	Yes	2.388 *	1.531–3.725

Note: 2 log likelihood = 598.115, chi-square = 186.41, * = *p* < 0.005.

## Data Availability

The raw data supporting the conclusions of this article will be made available by the authors on request.
